# Impact of environmental temperature on the survival outcomes of breast cancer: A SEER-based study

**DOI:** 10.1007/s10549-024-07369-9

**Published:** 2024-05-20

**Authors:** Ashish Gupta, Arya Mariam Roy, Kush Gupta, Kristopher Attwood, Asha Gandhi, Stephen Edge, Kazuaki Takabe, Elizabeth Repasky, Song Yao, Shipra Gandhi

**Affiliations:** 1 Department of Medicine, Roswell Park Comprehensive Cancer Center, Elm and Carlton Streets, Buffalo, NY 14203 USA; 2 Department of Medicine, University of Massachusetts Medical School–Baystate, Springfield, MA USA; 3 Department of Biostatistics and Bioinformatics, Roswell Park Comprehensive Cancer Center, 665 Elm Street, Buffalo, NY 14203 USA; 4 Department of Physiology, SGT University, Ghaziabad, India; 5 Department of Surgical Oncology, Roswell Park Comprehensive Cancer Center, Elm and Carlton Streets, Buffalo, NY 14203 USA; 6 Department of Immunology, Roswell Park Comprehensive Cancer Center, 665 Elm Street, Buffalo, NY 14203 USA; 7 Department of Cancer Prevention and Control, Roswell Park Comprehensive Cancer Center, 665 Elm Street, Buffalo, NY 14203 USA

**Keywords:** Breast Cancer, Environmental temperature, Cold stress, Overall survival

## Abstract

**Background:**

Experimental evidence in tumor-bearing mouse models shows that exposure to cool, that is, sub-thermoneutral environmental temperature is associated with a higher tumor growth rate and an immunosuppressive tumor immune microenvironment than seen at thermoneutral temperatures. However, the translational significance of these findings in humans is unclear. We hypothesized that breast cancer patients living in warmer climates will have better survival outcomes than patients living in colder climates.

**Methods:**

A retrospective population-based analysis was conducted on 270,496 stage I-III breast cancer patients, who were retrieved from the Surveillance, Epidemiology and End Results (SEER) over the period from 1996 to 2017. The average annual temperature (AAT) was calculated based on city level data from the National Centers for Environmental Information.

**Results:**

A total of 270, 496 patients were analyzed. Temperature as assessed in quartiles. After adjusting for potential confounders, patients who lived in the 3rd and 4th quartile temperature regions with AAT 56.7–62.5°F (3rd quartile) and > 62.5°F (4th quartile) had a 7% increase in the OS compared to patients living at AAT < 48.5°F (1st quartile) (HR 0.93, 95% CI 0.90–0.95 and HR 0.93, 95% CI 0.91–0.96, respectively). For DSS, When comparing AAT quartiles, patients living with AAT in the range of 56.7–62.5°F and > 62.5°F demonstrated a 7% increase each in DSS after adjustment (HR 0.93, 95% CI 0.90–0.96 and HR 0.93, 95% CI 0.90–0.96).

**Conclusions:**

Higher environmental temperatures are associated with significantly better OS and DSS in breast cancer patients. Future research is warranted to confirm this observation using large datasets to elucidate the underlying mechanisms and investigate novel therapeutic strategies to minimize this geographic disparity in clinical outcomes.

**Supplementary Information:**

The online version contains supplementary material available at 10.1007/s10549-024-07369-9.

## Background

The 2024 cancer statistics estimated 313,510 new cases of invasive breast cancer and 42,780 related deaths in the United States (US) [1]. The survival outcomes of breast cancer have undergone a paradigm shift in recent decades, with a reported 5-year survival of > 85% in many high-income countries [2]. Nonetheless, there are global and regional disparities in breast cancer survival outcomes, largely attributed to treatment availability, screening modalities, and the quality of care [3]. Recently, studies have identified geographical disparities in the survival trends of breast cancer and efficacy of treatment modalities within the same country or healthcare system, which cannot be solely attributed to race, ethnicity or other patient-specific factors [4].

Several environmental, lifestyle, and metabolic factors promote tumorigenesis through genetic alterations, epigenetic changes, induction of pro-tumorigenic signaling pathways, and suppression of pro-apoptotic signaling [5–7]. Recent experimental evidence highlighted significant interactions between thermal stress, i.e., cool ambient housing temperature, and cancer progression, where stress responses and hormones were found to mediate molecular mechanisms linked to an immunosuppressive tumor microenvironment (TME) [8]. Kokolus et al. showed an association between sub-thermoneutral housing temperature (22 °C) and tumor growth rate in tumor-bearing 4T1 murine models. Compared to sub-thermoneutral temperature, mice living under thermoneutral housing temperature (30 °C) showed significant reductions in tumor growth rate and metastasis; tumor exhibited a more favorable TME with more antitumor CD8^+^T-cells and fewer pro-cancerous myeloid-derived suppressor cells (MDSCs) and regulatory T-cells (Tregs) [9–11].

Epidemiological data suggest that countries with the coldest temperatures have the highest cancer incidence. Thus, a "cancer-cold" hypothesis was advocated by researchers, which proposes a significant association between low temperature and increased incidence of malignancy [12]. Similarly, a US population-based study found that people living in cold climate counties may have a higher cancer incidence, including breast cancer, while controlling for confounding variables such as ethnicity, age, gender or income [13], Sharma et al. reported that the incidence of 13 cancer types including breast cancer (out of the 16 studied cancers) was negatively correlated with the average annual temperature (AAT) [14].

Furthermore, Sharma et al. also reported that patients with cancer living in countries with a higher AAT had higher cancer-related mortality compared to countries with a lower AAT. Many factors such as lifestyle, social situation, healthcare system, reporting mechanisms etc., may affect the mortality rates across countries. In addition, the temperature range within a country can be highly variable [15]. Nonetheless, the prognostic significance of environmental temperature on breast cancer survival has not been studied yet. We hypothesized that breast cancer patients living in warmer climates in the US have better OS and DSS compared to patients living in colder climates.

## Methods

### Study design and data retrieval

A retrospective population-based analysis was conducted utilizing the Surveillance, Epidemiology and End Results (SEER) Database. The SEER database is from the National Cancer Institute (NCI), covering approximately 35% of the US population.

### Temperature data

Temperature data were obtained at the county level from the National Centers for Environmental Information (NCEI; https://www.ncei.noaa.gov/) data portal from 1996 to 2017. The NCEI is a US government agency that provides access to environmental data, including average temperature data by county, month, and year [16]. The NCEI collects temperature data from a variety of sources, including weather stations, buoys, ships, and satellites. The temperature data is collected using a variety of instruments and techniques, depending on the type of data source. For example, data from weather stations are typically collected using a thermometer. For satellites, temperature data are collected by measuring the infrared radiation emitted by the earth's surface and atmosphere. The temperature data is then calculated using algorithms that consider factors such as atmospheric conditions and surface emissivity [16].

For each month during the period from 1996 to 2017, the AAT of a county was calculated as the average temperature across the preceding and succeeding six months. For example, the AAT for January 2010 is calculated as the average monthly temperature from July 2009 (preceding six months) to July 2010 (succeeding six months). The AAT data were then merged with the SEER patient data by county (FIPS code) and year and month of breast cancer diagnosis.

### County level education and unemployment rate data

County-level education and unemployment data were obtained from the Economic Research Service of the U.S. Department of Agriculture (https://www.ers.usda.gov/data-products/county-level-data-sets/). Two education variables were generated using the available data: 1) % of adult within a county with at least a high school education, and b) % of adults within a county that completed a college degree. The education data was collected for the years 1990, 2000, and 2008–2012. For years in between, the carry-forward imputation method was used. For example, the 1990 values were used in 1996, 1997, 1998, and 1999. The unemployment rate for a county during a given year was defined as 100 × number unemployed annual average/Civilian labor force annual average. The education and unemployment data were merged with the SEER patient data by county (FIPS code) and year of diagnosis. FIPS codes or Federal Information Processing Standards Codes are unique codes assigned one per county. Institutional Review Board approval was waived as the SEER database is publicly available and de-identified**.**

### Study population

We retrieved data of all adult patients with stage I-III breast cancer who were diagnosed from 1996 to 2017. We identified the site of malignancy through the 3rd edition of the WHO International Classification of Diseases for Oncology. Patients were excluded if they had missing information on staging, OS, and residence.

### Statistical analysis

OS is the time from diagnosis until all cause mortality or last follow-up. DSS is the time from diagnosis until cancer-specific mortality or last follow-up (death due to other causes are censored). OS and DSS were summarized using Kaplan–Meier methods. Optimal thresholds for the AAT to identify worse vs better survival outcomes were obtained using the maximal log-rank approach. The maximal log-rank test identifies the univariate temperature threshold that produces the largest chi-square statistic associated with the log-rank test comparing observations above and below a given threshold [17]. Patient demographic and clinical characteristics were summarized by the categorized AAT (categories defined by the quartiles), with comparisons made using the Kruskal–Wallis and chi-square tests, as appropriate. Univariate associations between AAT and survival outcomes were evaluated using the Cox regression model. The multivariable Cox regression model was used to evaluate the association of survival with AAT while adjusting for additional covariates, which were identified using the backward selection approach (alpha exit = 0.10). The candidate variables included age, race, ethnicity, marital status, rural or urban area, breast cancer subtype, laterality, tumor stage and grade, type of treatment received in the neoadjuvant/adjuvant setting, year of diagnosis, county education levels, and county unemployment rates. Additional analyses examined the potential interaction between the effects of temperature and race on survival. A Cox regression model was fit with temperature, race, and their two-way interaction as predictors; where the overall test about the interaction term is of interest. All model assumptions were verified visually, using the appropriate graphical summaries of model residuals. Hazard ratios (HR) corresponding to AAT were obtained from model estimates and presented with a 95% confidence interval (CI). Subgroup analyses were performed according to the breast cancer subtype. All analyses were conducted in SAS v9.4 (Cary, NC) at a two-sided significance level of 0.05.

## Results

### Characteristics of study cohort

**Fig. 1:**
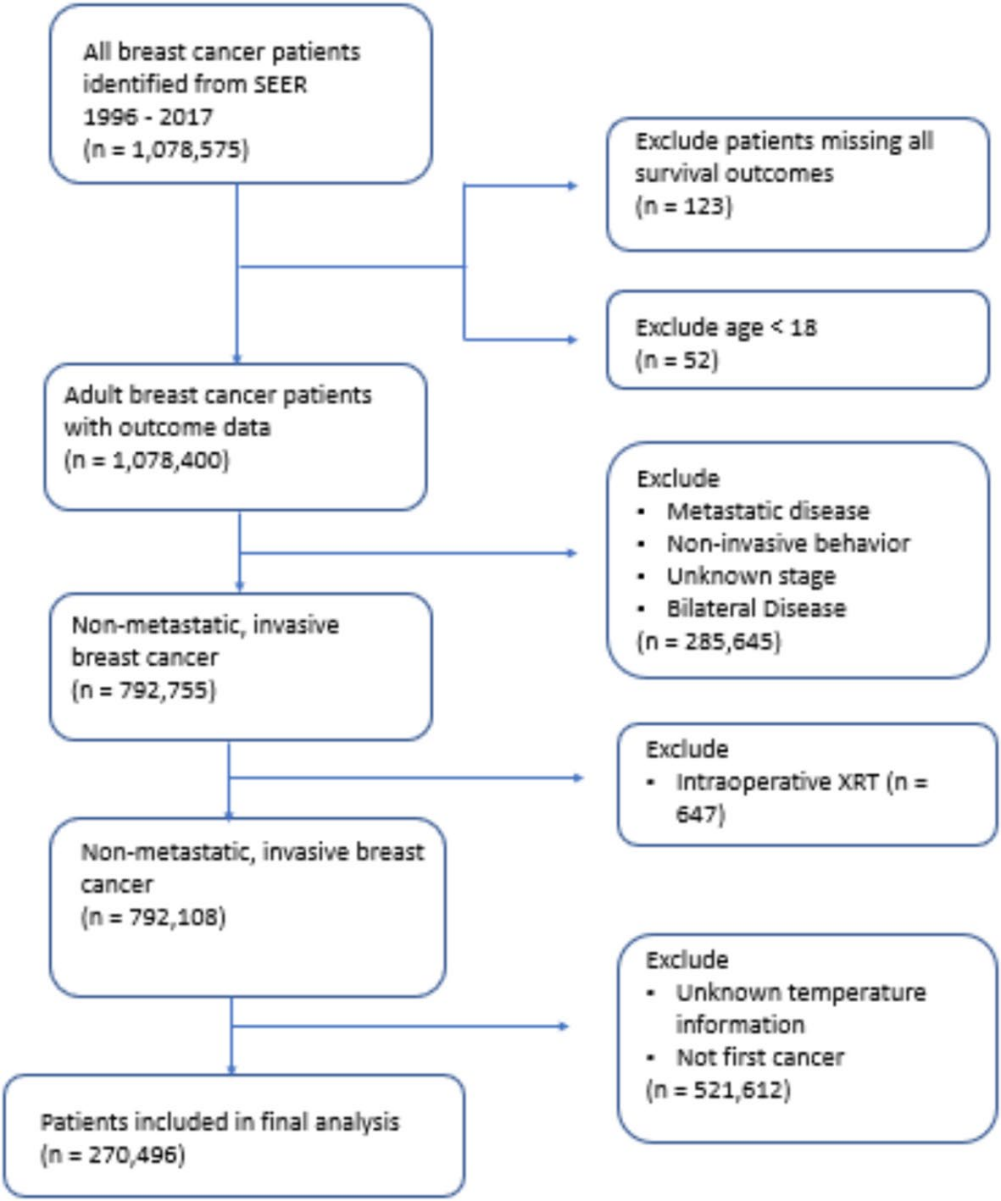
Study flowchart of patient selection

Data of 792,755 adult patients with non-metastatic invasive unilateral breast cancer with available survival outcomes were identified from SEER 1996 – 2017 database. Data of 522,259 patients were excluded as they received intraoperative radiotherapy,did not have temperature data, or breast cancer was not the first malignancy. A total of 270,496 patients were analyzed (Fig. 1). Overall, 77.9% of the patients were white, and 62.6% were > 55 years old. Receptor status data were available for 32.5% of patients. Among those with available data, 69.6% were HR + /HER2-, 13.3% triple-negative breast cancer (TNBC), 11.9% HR + /HER2 + , and 5.2% HR-/HER2 + . Nearly, 43.4% received chemotherapy, and 94.2% had surgery.

Table 1 shows the baseline characteristics of patients according to the AAT quartiles.

**Table 1:** Baseline characteristics of the included patients according to an AAT Quartiles

		** < 48.5°F**	**48.5–56.7 °F**	**56.7–62.5 °F**	** > 62.5 °F**	**P-value**
Overall	N	67,687 (25.0)	67,567 (25.0)	67,661 (25.0)	67,581 (25.0)	
Age at Diagnosis (in years)	Mean/Std/N	59.7/13.6/67686	59.4/13.6/67567	60.1/13.8/67658	60.4/14.0/67581	< .001
	Median/Min/Max	59.0/18.0/114.0	59.0/17.0/107.0	60.0/18.0/104.0	60.0/17.0/108.0	
Age (in years)	< 45	9,229 (13.6%)	9,564 (14.2%)	9,117 (13.5%)	8,918 (13.2%)	< .001
	45–55	16,161 (23.9%)	16,600 (24.6%)	16,271 (24.0%)	15,939 (23.6%)	
	55–65	17,506 (25.9%)	17,354 (25.7%)	16,742 (24.7%)	16,446 (24.3%)	
	> = 65	24,790 (36.6%)	24,049 (35.6%)	25,528 (37.7%)	26,278 (38.9%)	
Sex	Male	434 (0.6%)	417 (0.6%)	539 (0.8%)	521 (0.8%)	< .001
	Female	67,253 (99.4%)	67,150 (99.4%)	67,122 (99.2%)	67,060 (99.2%)	
Race	White	55,190 (81.5%)	50,556 (74.8%)	50,339 (74.4%)	55,504 (82.1%)	< .001
	Black	10,664 (15.8%)	9633 (14.3%)	8667 (12.8%)	10,287 (15.2%)	
	Other	1,833 (2.7%)	7378 (10.9%)	8655 (12.8%)	1790 (2.6%)	
Ethnicity	Non-Hispanic	63,423 (93.7%)	62,603 (92.7%)	62,961 (93.1%)	63,866 (94.5%)	< .001
	Hispanic	4264 (6.3%)	4964 (7.3%)	4700 (6.9%)	3715 (5.5%)	
Insurance	Not Insured	836 (1.2%)	580 (0.9%)	1066 (1.6%)	1078 (1.6%)	< .001
	Private Insurance	38,253 (56.5%)	35,457 (52.5%)	37,747 (55.8%)	34,931 (51.7%)	
	Government	28,598 (42.3%)	31,530 (46.7%)	28,848 (42.6%)	31,572 (46.7%)	
Urban/Rural	Urban	15,131 (22.4%)	9408 (13.9%)	2590 (3.8%)	5531 (8.2%)	< .001
	Rural	52,556 (77.6%)	58,159 (86.1%)	65,071 (96.2%)	62,050 (91.8%)	
Married	No	30,455 (45.0%)	28,965 (42.9%)	30,196 (44.6%)	30,504 (45.1%)	< .001
	Yes	37,232 (55.0%)	38,602 (57.1%)	37,465 (55.4%)	37,077 (54.9%)	
Grade	I/II	40,459 (59.8%)	40,809 (60.4%)	38,072 (56.3%)	38,873 (57.5%)	< .001
	III	23,707 (35.0%)	22,848 (33.8%)	24,428 (36.1%)	23,258 (34.4%)	
	Unknown	3,521 (5.2%)	3,910 (5.8%)	5,161 (7.6%)	5,450 (8.1%)	
Clin Stage	1	33,204 (49.1%)	33,783 (50.0%)	33,593 (49.6%)	34,428 (50.9%)	< .001
	2	25,200 (37.2%)	24,720 (36.6%)	24,893 (36.8%)	24,287 (35.9%)	
	3	9283 (13.7%)	9064 (13.4%)	9175 (13.6%)	8866 (13.1%)	
Sub-Type	TNBC	2960 (4.4%)	2810 (4.2%)	2890 (4.3%)	3003 (4.4%)	< .001
	HR + /HER2 +	2571 (3.8%)	2725 (4.0%)	2601 (3.8%)	2609 (3.9%)	
	HR-/HER +	1082 (1.6%)	1141 (1.7%)	1156 (1.7%)	1139 (1.7%)	
	HR + /HER2-	14,682 (21.7%)	15,371 (22.7%)	15,657 (23.1%)	15,370 (22.7%)	
	Not Reported	46,392 (68.5%)	45,520 (67.4%)	45,357 (67.0%)	45,460 (67.3%)	
Laterality	right	33,445 (49.4%)	33,284 (49.3%)	33,285 (49.2%)	33,268 (49.2%)	0.862
	left	34,242 (50.6%)	34,283 (50.7%)	34,376 (50.8%)	34,313 (50.8%)	
Surgery	No	1952 (2.9%)	1832 (2.7%)	2054 (3.0%)	2056 (3.0%)	< .001
	Yes	65,615 (96.9%)	65,697 (97.2%)	65,505 (96.8%)	65,438 (96.8%)	
	Not Reported	120 (0.2%)	38 (0.1%)	102 (0.2%)	87 (0.1%)	
Radiation	None	32,881 (48.6%)	30,543 (45.2%)	32,232 (47.6%)	33,726 (49.9%)	< .001
	Yes	34,541 (51.0%)	36,559 (54.1%)	34,792 (51.4%)	32,983 (48.8%)	
	Not Reported	265 (0.4%)	465 (0.7%)	637 (0.9%)	872 (1.3%)	
Chemotherapy	None	37,780 (55.8%)	37,819 (56.0%)	38,633 (57.1%)	40,363 (59.7%)	< .001
	Yes	29,907 (44.2%)	29,748 (44.0%)	29,028 (42.9%)	27,218 (40.3%)	
County Unemployment %	Mean/Std/N	6.8/2.4/62446	6.5/2.5/60622	6.0/2.2/66371	6.2/2.2/60949	< .001
	Median/Min/Max	6.2/2.2/23.2	5.9/2.2/21.6	5.6/1.7/16.2	5.8/1.7/17.3	
County % with HS Degree	Mean/Std/N	83.0/7.9/67687	84.7/6.3/67567	85.5/6.3/67661	83.2/6.6/67581	< .001
	Median/Min/Max	85.0/49.5/96.5	86.2/39.4/96.5	86.5/59.4/95.7	83.8/53.3/95.7	
County % with College Degree	Mean/Std/N	26.8/11.5/67687	31.3/10.8/67567	31.7/9.8/67661	29.1/9.9/67581	< .001
	Median/Min/Max	26.7/4.9/55.9	34.1/5.3/56.1	31.5/7.4/56.1	28.7/7.4/55.8	

*HS* high school

Significant values are in bold

### Predictors of OS and DSS

The univariate analysis for OS revealed several significant factors. Factors such as: increase in age, Black patients, unemployment, had worse OS. Other tumor-related factors associated with worse OS: Grade III/IV, stage 2 and 3, left-sided breast cancer and TNBC. Factors such as: white race, private insurance, living in a metropolitan area, Stage 1 cancer, and HR + and HER2 + disease, receipt of treatment, Hispanic ethnicity were associated with better OS. (Supplementary Table 1A). Similar findings were noted in DSS except receipt of chemotherapy and Hispanic ethnicity which were associated with worse DSS (Supplementary Table 1B).

### The association between OS/DSS and AAT

**Fig. 2:**
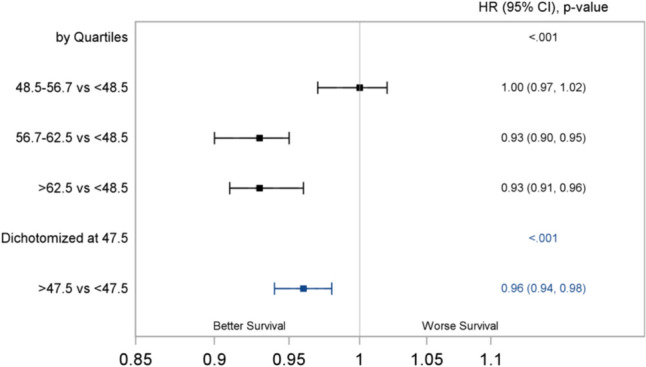
Forest Plot of the Adjusted Cox Regression Analysis for OS

**Fig. 3:**
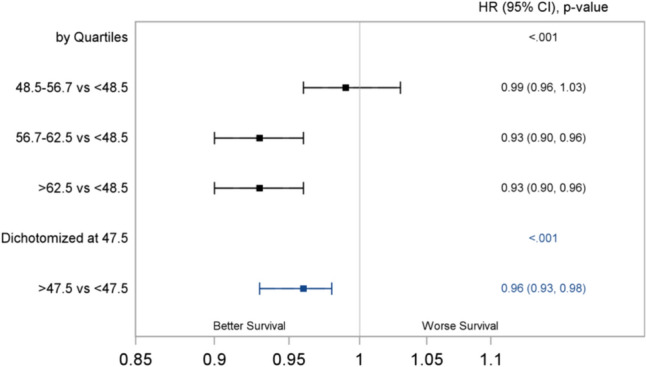
Forest Plot of the Adjusted Cox Regression Analysis for DSS

The median OS of the overall cohort was 227 (95% CI 225 – 230) months, that is 18.9 years. The median DSS of the overall cohort was not estimated. Figures 2 and 3 show the univariate and multivariable Cox regression analysis. Temperature was assessed in quartiles. After adjusting for potential confounders, who lived in the 3rd and 4th quartile temperature regions with AAT 56.7–62.5°F (3rd quartile) and > 62.5°F (4th quartile) had a 7% increase in the OS compared to patients living at AAT < 48.5°F (1st quartile) (HR 0.93, 95% CI 0.90–0.95 and HR 0.93, 95% CI 0.91–0.96, respectively). For DSS, when comparing AAT quartiles, patients living with AAT in the range of 56.7–62.5°F and > 62.5°F demonstrated a 7% increase each in DSS after adjustment (HR 0.93, 95% CI 0.90–0.96 and HR 0.93, 95% CI 0.90–0.96).

Optimal threshold for the AAT by maximal log-rank approach was 47.5°F. We also analyzed the data with a different cut-off of 56.7°F which provided a lower log-rank chi square statistic than that of 47.5°F which justified the selection of 47.5°F as the optimal threshold for AAT (Supplementary Table 2). Compared to those living in regions with AAT ≤ 47.5°F, patients living at AAT ≥ 47.5°F had a a 4% increase in the OS (HR 0.96, 95% CI 0.94–0.98, p < 0.001) and 4% increase in DSS (HR 0.96, 95% CI 0.93–0.98) on multivariate analysis.

### OSS and DSS by AAT by subtypes

The subgroup analysis showed that, compared to patients living with AAT ≤ 47.5°F, patients diagnosed with the TNBC subtype had a 10% increase in OS in in the adjusted models (HR 0.90, 95% CI 0.81–0.99, p = 0.044). For the HER2 + HR- subtype, there was a 24% increase after adjustment (HR 0.76, 95% CI 0.62–0.93, p = 0.008). Likewise, the adjusted models showed 12% and 21% increases in the OS in patients with HR + HER2- and HR + HER2 + subtypes (Table 2).

**Table 2:** Univariate and Multivariate Analysis of AAT as a predictor of OS and DSS

Variable		OS			DSS		
		Unadjusted HR (95% CI	Adjusted HR (95% CI)	P-value	Unadjusted HR (95% CI	Adjusted HR (95% CI)	P-value
Subtypes (Ref AAT ≤ 47.5)	TNBC > 47.5	0.91 (0.83, 1.01)	0.90 (0.81, 0.99)	0.044	0.92 (0.81, 1.03)	0.89 (0.79, 1.00)	0.051
	HER2 + HR- > 47.5	0.71 (0.58, 0.87)	0.76 (0.62, 0.93)	0.008	0.68 (0.53, 0.86)	0.72 (0.56, 0.92)	0.008
	HR + HER2- > 47.5	0.83 (0.77, 0.89)	0.88 (0.82, 0.95)	< 0.001	0.86 (0.77, 0.96)	0.86 (0.76, 0.96)	0.008
	HR + HER2 + > 47.5	0.78 (0.66, 0.91)	0.79 (0.67, 0.94)	0.006	0.65 (0.53, 0.80)	0.68 (0.55, 0.84)	< 0.001

HER2 + HR- patients had a 32% and 28% increase in unadjusted (HR 0.68, 95% CI 0.53–0.86) and adjusted DSS (HR 0.72, 95% CI 0.56–0.92, p = 0.008), respectively. Significant increases in the DSS were also noted in the HR + HER2- and HR + HER2 + subtypes (Table 2).

### Association of race, other covariates with temperature and survival

Race is thought to influence responses to different temperatures.With regard to the interactions between temperature and race, for OS, the white and black cohorts have a similar temperature-survival profile. In the “other” race cohort, benefit is only observed at the extreme end of high temperatures (HR 0.76, 95% CI 0.62–0.93; p = 0.002; Table 3).

**Table 3:** Race-Temperature Interactions

Outcome	Race	Temp HR (95% CI)				Within Race p-value	Interaction p-value
		** ≤ 48.5**	**48.5–56.7**	**56.7–62.5**	** > 62.5**		
OS	White	Ref	0.99 (0.97, 1.02)	0.91 (0.89, 0.94)	0.92 (0.90, 0.95)	< 0.001	< 0.001
	Black	Ref	1.02 (0.96, 1.08)	0.92 (0.87, 0.97)	0.99 (0.94, 1.05)	0.036	
	Other	Ref	1.05 (0.91, 1.21)	1.09 (0.95, 1.26)	0.76 (0.62, 0.93)	0.002	
DSS	White	Ref	0.98 (0.94, 1.02)	0.91 (0.87, 0.95)	0.90 (0.86, 0.94)	< 0.001	0.006
	Black	Ref	1.03 (0.96, 1.11)	1.00 (0.93, 1.07)	1.04 (0.97, 1.12)	0.19	
	Other	Ref	0.96 (0.80, 1.16)	0.91 (0.76, 1.10)	0.74 (0.57, 0.95)	0.11	

Significant values are in bold

For DSS, the white cohort has a temperature-survival profile similar to what is observed in the combined data. In the “other” race cohort, benefit is only observed at the extreme end of high temperatures. And for the black cohort, no significant association is observed. (Table 3).

Since availability of treatment may also impact survival, we analyzed the interaction of insurance types and type treatment (surgery/chemotherapy/radiation) with temperature and survival and observed the same association that warmer areas are associated with better survival (Supplementary Table 3).

## Discussion

Ambient temperature is an important driver of human health, with well-established associations between ambient temperature changes and disease burden [18], mortality [19], and morbidity [20]. Preclinical and epidemiologic data have suggested an association between cancer progression and climate. Our analysis demonstrates that higher AAT was an independent predictor of improvement in OS and DSS in stage I-III breast cancer patients. We found that patients living at AAT ≥ 47.5°F had a 4% improvement in OS and a 4% improvement in DSS A similar relationship was seen when assessed by quartiles with a 7% improvement in OS and DSS in the 3rd and 4th quartiles of temperature (AAT 56.7–62.5°F and > 62.5°F respectively). This improvement in survival was also consistently observed among different breast cancer subtypes. Such findings indicate the significant impact of ambient temperature on the survival of breast cancer patients. A greater impact at more extremes of temperature may indicate that the effect of temperature on breast cancer survival is not linear. The findings that a warmer climate is associated with favorable survival outcomes in stage I-III breast cancer patients is in line with our recent publication showing significantly worse DSS and an immunosuppressive TME in breast cancer patients whose tumors have high thermogenesis scores (reflective of exposure to cold weather) [21]. This is also in line with data recently reported from gastroesophageal adenocarcinoma showing that higher environmental tempeartures are associated with improved OS and DSS[22].

To the best of our knowledge, this is the first study assessing the impact of environmental temperature on overall survival (OS) and disease-specific survival (DSS) in stage I-III breast cancer patients in the US.

Interestingly, our study showed an improvement in overall survival with increasing temperature both among Black and White races, but an improvement in DSS was only observed for White but not for Black patients. Disease specific survival is a better estimate over OS when determining prognosis as it reflects survival related to the underlying diagnosis. This could reflect the aggressive biology of breast cancer among Black patients.

While the exact mechanisms and pathways underpinning the negative impact of cold climate on oncological outcomes have not been fully elucidated yet, we propose several explanations for these findings. We hypothesize that impaired antitumor immune responses in the TME under cold stress contribute to worse outcomes among patients living in a colder climate. As previously mentioned, sub-thermoneutral temperatures were associated with impaired immune response in the TME. Housing mice under these temperatures resulted in a lower number of functional antitumor CD8^+^ T and overexpression of MDSCs [23], a potent suppressor of antitumor immune responses and CD8 + T proliferation [23, 24]. Besides, Bucsek et al. found Tregs and programmed death receptor-1 (PD-1) overexpression in mice exposed to cold stress. C [25].

Our study findings that validate the association between temperature and outcomes in breast cancer have clinical and translational implications. Our study validates preclinical data results, noting an association between tumor behavior and temperature. It was also shown that housing temperature alters mouse biology and antitumor immune responses and sensitivity of tumors to therapeutic interventions [8]. Therefore, the housing temperature of cancer models should be considered while assessing therapeutic interventions, alongside considering climate as a possible confounder during the clinical assessment of novel therapies. Building on this knowledge of immunosuppression in cold temperatures, targeted interventions for neural thermoreceptive pathways are proposed to improve clinical outcomes amongst colder patients. In a phase I clinical trial, adding a non-selective β-blocker to pembrolizumab, an immune checkpoint inhibitor, led to an impressive objective response rate of 78% in patients with metastatic melanoma [26]. In breast cancer models, non-selective β-blocker reversed the effects of cold stress on tumor growth and spread [27]. In a phase II clinical trial, 60 breast cancer patients were randomized to receive propranolol or placebo one week preoperatively. The results showed that propranolol improved the antitumor immune response and reduced biomarkers associated with metastatic potential [28].

The drivers of tumorigenesis and alteration in the TME in those who live in colder environments can be analyzed in detail using metabolomics which involves the measurements of various small molecule metabolites, including signaling mediators, nutrients, proteins in the blood, and the metabolic products of these molecules in the body fluids [29]. This would help us to develop personalized treatment options for those with aggressive malignancies who live in colder environments. As cold therapy for alopecia [30] and cryotherapy to prevent chemotherapy-induced neuropathy is gaining momentum [31], the clinical outcomes of patients who undergo these treatments are worth examining to scrutinize the possible impact on their clinical outcomes with these novel interventions. In recent years, treatments involving hyperthermia have been utilized with success in multiple cancers. Hyperthermia, when combined with chemotherapy and immunotherapy, has shown promising results in gynecological malignancies treatments, including Hyperthermic Intraperitoneal Chemotherapy (HIPEC) [32] and has increased therapeutic effectiveness in melanoma when combined with radiation therapy [33]. Considering the findings from our study that breast cancer patients residing in warm climatic regions demonstrate a better prognosis, we may explore the potential of hyperthermia treatments for breast cancer in the future, although further investigation is needed to confirm its efficacy in light of limitations of the study below.

In light of the observations made in our study, it is imperative to consider the broader implications of ambient temperature on OS, encompassing both cancer-specific and non-cancer mortality. Our analysis suggests that the association of ambient temperature with OS mirrors its relation to cancer survival, hinting at a significant impact of temperature on non-cancer mortality as well. This finding necessitates a deeper exploration into how ambient temperatures might influence health outcomes beyond cancer progression. Factors such as environmental stress, the exacerbation of chronic conditions, and general health vulnerabilities in varying temperatures could also play a role. These insights prompt us to think beyond the traditional scope of tumor biology and consider a more integrated approach, where environmental factors are acknowledged as influential determinants of patient health and survival. This perspective broadens our understanding of the intricate relationship between environmental conditions and health outcomes and underscores the need for comprehensive patient care strategies that account for such environmental influences.

Our study has the strengths of a large sample size and representation of the US population. Our findings aligned with our preclinical studies, showing a significant association between ambient temperature and TME. Nonetheless, the study has several limitations. First, the analysis is based on retrospective data, which limits the control of the outcomes. Breast cancer subtype data were missing in a considerable proportion of the SEER cohort. In terms of AAT, patients may have changed their residence during the course of surgery and follow-up, which can influence our results, and, therefore, a time-dependent analysis might not be as informative. Since this is an ecological study examining the relationship between clinical outcome and temperature exposure at a population level, individual-level data (e.g., air-conditioning, personal and lifestyle factors including dietary intake, physical activity (patients from colder areas may do less outdoor activities vs those in warmer temperatures), Vitamin D levels were not available. In addition, variables including socioeconomic status, average family incomes, access to high-quality healthcare, time to first treatment, post-diagnosis weight change are not available in SEER, so were not able to be adjusted for. Unlike mice, humans can mitigate cold stress by changing their environment. For example, some people are more exposed to the effects of ambient temperature, air pollution, and light, e.g., construction workers, vs others who spend the majority of their time in a temperature-controlled setting, e.g., desk workers, and we are not able to account for this disparity in our analysis.

## Conclusions

In conclusion, higher environmental temperatures are associated with significantly better survival outcomes in stage I-III breast cancer patients. Considering the limitations of the current study, future research is warranted to confirm this observation using center-specific and/or prospective studies, which would provide more detailed data about monthly temperature and patients' whereabouts to consider a time-dependent study model and help us dissect data to understand the underlying mechanisms and develop strategies to address this geographic disparity in clinical outcomes.

### Supplementary Information

Below is the link to the electronic supplementary material.

Supplementary file1 (DOCX 31 KB)

## Data Availability

All data utilized in this article is available in public datasets – SEER. Data analyzed during this study are included in this published article and its Appendix.

## Abbreviations

AAT: Average annual temperature
C: Celsius
CDCC: Charlson/deyo comorbidity score
CI: Confidence interval
DSS: Disease specific survival
F: Fahrenheit
HER2: Human epidermal growth factor receptor 2
HIPEC: Hyperthermic intraperitoneal chemotherapy
HR: Hazard ratio
HR: Hormone receptor
MDSCs: Myeloid derived suppressor cells
NCEI: National centers for environmental information
NCI: National cancer institute
OR: Odds ratio
OS: Overall survival
PD-1: Programmed death receptor-1
SEER: Surveillance, epidemiology and end results
T-regs: Regulatory T-cells
TME: Tumor microenvironment
TNBC: Triple negative breast cancer
US: United States
