# Supplementary material for: Impact of environmental temperature on the survival outcomes of breast cancer: A SEER-based study
Source: Breast Cancer Res Treat. 2024 May 20;207(2):383–92. doi: 10.1007/s10549-024-07369-9 (PMC11297050; doi:10.1007/s10549-024-07369-9)
Supplement: Supplementary file 1 — Supplementary file1 (DOCX 31 KB) [file 10549_2024_7369_MOESM1_ESM.docx]

**Supplementary Table 1A: Univariate Analysis for OS**

| **Variable** |  | **Hazard Ratio (95% CI)** | **p-value** |
| --- | --- | --- | --- |
| Age at Diagnosis | Unit Inc. | 1.05 (1.05, 1.06) | <.001 |
| Age | <45 | 1.00 | <.001 |
|  | 45-55 | 0.85 (0.82, 0.88) |  |
|  | 55-65 | 1.14 (1.10, 1.17) |  |
|  | >=65 | 3.20 (3.12, 3.29) |  |
| Sex | Male | 1.00 | <.001 |
|  | Female | 0.55 (0.52, 0.60) |  |
| Race | White | 1.00 | <.001 |
|  | Black | 1.35 (1.32, 1.37) |  |
|  | Other | 0.67 (0.65, 0.70) |  |
| Ethnicity | Non-Hispanic | 1.00 | <.001 |
|  | Hispanic | 0.90 (0.87, 0.93) |  |
| Insurance | Not Insured | 1.00 | <.001 |
|  | Private Insurance | 0.78 (0.72, 0.85) |  |
|  | Government | 0.93 (0.86, 1.01) |  |
| Urban/Rural | non-Metro | 1.00 | <.001 |
|  | Metro | 0.81 (0.79, 0.83) |  |
| Married | No | 1.00 | <.001 |
|  | Yes | 0.52 (0.51, 0.53) |  |
| Grade | I/II | 1.00 | <.001 |
|  | III/IV | 1.41 (1.38, 1.43) |  |
|  | Not Reported | 1.30 (1.26, 1.34) |  |
| Clin Stage | 1 | 1.00 | <.001 |
|  | 2 | 1.51 (1.49, 1.54) |  |
|  | 3 | 3.33 (3.27, 3.40) |  |
| Sub-Type | TNBC | 1.00 | <.001 |
|  | HR+/HER2+ | 0.42 (0.38, 0.45) |  |
|  | HR-/HER+ | 0.62 (0.56, 0.68) |  |
|  | HR+/HER2- | 0.38 (0.36, 0.40) |  |
| Laterality | Right | 1.00 | <.001 |
|  | Left | 1.03 (1.01, 1.04) |  |
| Surgery | No | 1.00 | <.001 |
|  | Yes | 0.26 (0.25, 0.27) |  |
|  | Not Reported | 0.73 (0.62, 0.88) |  |
| Radiation | None | 1.00 | <.001 |
|  | Yes | 0.59 (0.58, 0.60) |  |
|  | Not Reported | 0.80 (0.74, 0.87) |  |
| Chemotherapy | None | 1.00 | <.001 |
|  | Yes | 0.78 (0.77, 0.79) |  |
| County Unemployment % | Unit Inc. | 1.01 (1.00, 1.01) | <.001 |
| County % with HS Degree | Unit Inc. | 0.98 (0.98, 0.98) | <.001 |
| County % with College Degree | Unit Inc. | 0.99 (0.98, 0.99) | <.001 |

**Supplementary Table 1B: Univariate Analysis for DSS**

| **Variable** |  | **Hazard Ratio (95% CI)** | **p-value** |
| --- | --- | --- | --- |
| Age at Diagnosis | Unit Inc. | 1.01 (1.01, 1.01) | <.001 |
| Age | <45 | 1.00 | <.001 |
|  | 45-55 | 0.73 (0.71, 0.76) |  |
|  | 55-65 | 0.74 (0.71, 0.76) |  |
|  | >=65 | 1.04 (1.00, 1.07) |  |
| Sex | Male | 1.00 | <.001 |
|  | Female | 0.68 (0.60, 0.76) |  |
| Race | White | 1.00 | <.001 |
|  | Black | 1.84 (1.79, 1.89) |  |
|  | Other | 0.77 (0.73, 0.81) |  |
| Ethnicity | Non-Hispanic | 1.00 | 0.010 |
|  | Hispanic | 1.06 (1.01, 1.11) |  |
| Insurance | Not Insured | 1.00 | <.001 |
|  | Private Insurance | 0.53 (0.48, 0.58) |  |
|  | Government | 0.68 (0.61, 0.74) |  |
| Urban/Rural | non-Metro | 1.00 | <.001 |
|  | Metro | 0.85 (0.83, 0.88) |  |
| Married | No | 1.00 | <.001 |
|  | Yes | 0.66 (0.64, 0.67) |  |
| Grade | I/II | 1.00 | <.001 |
|  | III/IV | 2.62 (2.56, 2.68) |  |
|  | Not Reported | 1.75 (1.67, 1.83) |  |
| Clin Stage | 1 | 1.00 | <.001 |
|  | 2 | 3.08 (2.98, 3.17) |  |
|  | 3 | 10.11 (9.80, 10.43) |  |
| Sub-Type | TNBC | 1.00 | <.001 |
|  | HR+/HER2+ | 0.32 (0.29, 0.36) |  |
|  | HR-/HER+ | 0.59 (0.52, 0.66) |  |
|  | HR+/HER2- | 0.21 (0.20, 0.22) |  |
| Laterality | Right | 1.00 | 0.003 |
|  | Left | 1.03 (1.01, 1.06) |  |
| Surgery | No | 1.00 | <.001 |
|  | Yes | 0.20 (0.19, 0.21) |  |
|  | Not Reported | 0.74 (0.59, 0.92) |  |
| Radiation | None | 1.00 | <.001 |
|  | Yes | 0.66 (0.64, 0.67) |  |
|  | Not Reported | 0.94 (0.84, 1.05) |  |
| Chemotherapy | None | 1.00 | <.001 |
|  | Yes | 1.80 (1.76, 1.85) |  |
| County Unemployment % | Unit Inc. | 1.00 (1.00, 1.01) | 0.259 |
| County % with HS Degree | Unit Inc. | 0.98 (0.98, 0.98) | <.001 |
| County % with College Degree | Unit Inc. | 0.99 (0.99, 0.99) | <.001 |

Supplementary Table 2: Univariate and Multivariate Analysis using a AAT cut-off of 56.7

|  | OS | | DSS | |
| --- | --- | --- | --- | --- |
| **AAT ≤ 56.7** | Unadjusted HR (95% CI) | Adjusted HR (95% CI) | Unadjusted HR (95% CI) | Adjusted HR (95% CI) |
|  | 0.98 (0.97, 1.0) | 0.93 (0.92, 0.95) | 0.96 (0.94, 0.98) | 0.93 (0.91, 0.96) |

Supplementary Table 3: P-value for Interaction with Temperature (Quartiles)

|  | | |
| --- | --- | --- |
| **Covariate** | **OS** | **DSS** |
| Insurance | 0.003 | 0.68 |
| Chemotherapy | 0.065 | 0.21 |
| Radiation Therapy | 0.019 | 0.035 |
| Surgery | <0.001 | 0.027 |
